# Identification of genes and pathways associated with cytotoxic T lymphocyte infiltration of serous ovarian cancer

**DOI:** 10.1038/sj.bjc.6605820

**Published:** 2010-07-27

**Authors:** N Leffers, R S N Fehrmann, M J M Gooden, U R J Schulze, K A ten Hoor, H Hollema, H M Boezen, T Daemen, S de Jong, H W Nijman, A G J van der Zee

**Affiliations:** 1 Department of Gynaecologic Oncology (CB22), University Medical Center Groningen, University of Groningen, PO Box 30.001, Groningen 9700 RB, The Netherlands; 2 Department of Medical Oncology (DA13), University Medical Center Groningen, University of Groningen, PO Box 30.001, Groningen 9700 RB, The Netherlands; 3 Department of Genetics (DA13), University Medical Center Groningen, University of Groningen, PO Box 30.001, Groningen 9700 RB, The Netherlands; 4 Department of Medical Microbiology, Section of Molecular Virology (EB88), University Medical Center Groningen, University of Groningen, PO Box 30.001, Groningen 9700 RB, The Netherlands; 5 Department of Pathology and Medical Biology (EA10), University Medical Center Groningen, University of Groningen, PO Box 30.001, Groningen 9700 RB, The Netherlands; 6 Department of Epidemiology (FA40), University Medical Center Groningen, University of Groningen, PO Box 30.001, Groningen 9700 RB, The Netherlands

**Keywords:** gene, pathway, lymphocyte, ovarian cancer, microarray

## Abstract

**Background:**

Tumour-infiltrating lymphocytes (TILs) are predictors of disease-specific survival (DSS) in ovarian cancer. It is largely unknown what factors contribute to lymphocyte recruitment. Our aim was to evaluate genes and pathways contributing to infiltration of cytotoxic T lymphocytes (CTLs) in advanced-stage serous ovarian cancer.

**Methods:**

For this study global gene expression was compared between low TIL (*n*=25) and high TIL tumours (*n*=24). The differences in gene expression were evaluated using parametric *T*-testing. Selectively enriched biological pathways were identified with gene set enrichment analysis. Prognostic influence was validated in 157 late-stage serous ovarian cancer patients. Using immunohistochemistry, association of selected genes from identified pathways with CTL was validated.

**Results:**

The presence of CTL was associated with 320 genes and 23 pathways (*P*<0.05). In addition, 54 genes and 8 pathways were also associated with DSS in our validation cohort. Immunohistochemical evaluation showed strong correlations between MHC class I and II membrane expression, parts of the antigen processing and presentation pathway, and CTL recruitment.

**Conclusion:**

Gene expression profiling and pathway analyses are valuable tools to obtain more understanding of tumour characteristics influencing lymphocyte recruitment in advanced-stage serous ovarian cancer. Identified genes and pathways need to be further investigated for suitability as therapeutic targets.

Epithelial ovarian cancer is the most common cause of death from gynaecological malignancies (Parkin *et al*, 2005). The 5-year survival rates for ovarian cancer patients do not exceed 40%. This high mortality is best attributed to the absence of specific symptoms combined with the lack of reliable screening methods, which prevent diagnosis in the early stages of the disease in a majority of patients. Treatment generally consists of cytoreductive surgery followed by platinum- and taxane-containing chemotherapy. Classic prognostic factors are stage of disease at diagnosis, histological tumour type and grade, residual disease after primary surgery, and response to chemotherapy (Crijns *et al*, 2006). An increasing body of evidence suggests that next to these established prognostic factors, the presence of tumour-infiltrating lymphocytes (TILs) also independently contributes to prognosis (Zhang *et al*, 2003; Sato *et al*, 2005; Leffers *et al*, 2009a). Although the presence of TILs is generally considered a reflection of antitumour immunity, it is largely unknown why TILs are present in high numbers in some tumours and largely absent in others. It has been shown that endothelial factors and chemokines secreted by the tumour may have an important role (Curiel *et al*, 2004; Buckanovich *et al*, 2008). The existence of an antitumour immune repertoire in a selection of patients forms the rationale for the development of cancer immunotherapy. Although immunotherapy strategies generally induce potent peripheral immune responses in ovarian cancer patients, clinical responses have so far been disappointing (Hung *et al*, 2008). The combination of targeted agents that enhance lymphocyte recruitment to tumour sites with these immunotherapy strategies might be a lucrative approach to obtain clinical responses to immunotherapy. For instance, the *in vivo* addition of BQ-788, an endothelin B receptor antagonist, to previously immunogenic, but clinically ineffective, immunisation strategies resulted in enhanced homing of lymphocytes to tumours as well as improved clinical responses (Buckanovich *et al*, 2008).

To investigate what tumour factors contribute to the recruitment of lymphocytes, we analysed which genes and pathways were associated with the presence of tumour-infiltrating CTLs in a homogeneous group of 49 advanced-stage serous ovarian cancer patients previously profiled at our institute as part of a larger study (Crijns *et al*, 2009). The prognostic value of identified genes and pathways was subsequently validated on all 157 previously profiled late-stage serous ovarian cancer patients (Crijns *et al*, 2009). Furthermore, immunohistochemical staining of tissue microarrays was performed to validate findings.

## Patients and methods

### Patients

The presence of tumour-infiltrating T lymphocytes was previously evaluated by our group in 306 ovarian cancer patients (Leffers *et al*, 2009a). In short, we used a tissue microarray containing core biopsies (0.283 mm^2^ each) of tumour tissue obtained from each of 306 ovarian cancer patients at primary surgery. Sections were stained with anti-CD8 (1 : 20; Dako Cytomation, Glostrup, Denmark). The number of intraepithelial CD8^+^ CTLs were separately counted for each core and an average per core was calculated when at least two cores were present for a single tumour sample. Patients with >8 CD8^+^ T lymphocytes per 0.283 mm^2^ were found to have a better prognosis. For this study, we selected from this heterogeneous population only advanced-stage serous ovarian cancer patients with low (⩽5 per 0.283 mm^2^ of tumour) or high (⩾8 per 0.283 mm^2^ of tumour) CTL numbers who were also included in the previously published microarray study (Crijns *et al*, 2009). This large microarray study contained 157 advanced-stage ovarian cancer patients for whom fresh frozen tumour tissue was available. Only 63 of these patients were also represented on our tissue microarray, of whom only 49 met the requirements stipulated above.

Patients were treated at the University Medical Center Groningen by a gynaecological oncologist and staged according to FIGO classification (Cancer Committee of the International Federation of Gynaecology and Obstetrics, 1986). Tumours were graded and classified according to the WHO criteria by a gynaecological pathologist (Scully, 1999). Adjuvant chemotherapy generally consisted of different platinum-based treatment regimens. Response to chemotherapy was evaluated according to the WHO criteria (World Health Organization, 1979). After treatment, patients were followed-up for at least 10 years with gradually increasing intervals. Informed consent was obtained for the collection and storage of tumour samples in a tissue bank for future research. Information on clinicopathological characteristics and follow-up of patients was obtained from a computerised database in which information of all patients with epithelial ovarian cancer treated at our institute is prospectively recorded. For this study, relevant data were retrieved into a separate anonymous database. In this separate database, patient identity was protected by study-specific, unique patient codes. In case of uncertainties with respect to clinicopathological and follow-up data, the larger databases could only be checked through two data managers who have daily responsibilities for the larger database, thereby ascertaining the protection of patients’ identity. According to Dutch law no approval from our IRB was needed.

### Microarray analysis

As mentioned above, we selected 49 advanced-stage serous ovarian cancer patients based on the relative absence or presence of CD8^+^ TILs, who were previously profiled as part of a larger study (Crijns *et al*, 2009). In brief, after RNA extraction and amplification, samples were hybridised to two-colour 70-mer oligonucleotide microarrays (∼35 000 Operon v3.0 probes, Ebersberg, Germany). All samples were hybridised at least twice and samples were loaded using a random design to prevent systematic biases (Churchill, 2002; Kerr, 2003; Hsu *et al*, 2007). Arrays were scanned using the Affymetrix GMS428 (Affymetrix, Santa Clara, CA, USA). Expression values were calculated using Bluefuse software (BlueGnome, Cambridge, UK). Operon v3.0 probe identifiers were converted to official gene symbols using probe annotations provided by The Netherlands Cancer Institute (NKI, Amsterdam, The netherlands). Only oligonucleotides specifically responding with a single hit on a gene during a BLAST search were used. Expression values of multiple probes targeting a single gene were averaged, resulting in a total of 15 909 distinct genes. Subsequently, expression data of the multiple hybridisations per tumour sample were averaged. Microarray data of the previous, larger study from which our patients were selected are available at http://www.ncbi.nlm.nih.gov/geo/ under number GSE13876.

### Class comparison between low TIL and high TIL samples

The BRB Array Tools 3.6.0 software package, developed by the Biometric Research Branch of the US National Cancer Institute, was used for class comparison between low-TIL and high-TIL samples. Differentially expressed genes were identified using a paired *T*-test (threshold *P*<0.05).

### Gene set enrichment analysis (GSEA)

As it is unclear whether large differences in the expression of a single gene are biologically more relevant than more subtle, although coordinated, differences in a set of genes belonging to a single biological pathway, we performed gene set enrichment analysis. Expression data of all 15 909 genes were compared against functional gene sets to determine whether any of these sets were enriched in samples containing high or low numbers of CD8^+^ TILs. The comparison was performed using 340 gene sets reported in two databases (174 sets from BIOCARTA: http://www.biocarta.com; 166 sets from Kyoto Encyclopedia of Genes and Genomes database (KEGG): http://www.genome.jp/kegg/). Statistical significance of enrichment was determined using an empirical gene-based permutation test using 1000 permutations. Gene sets with an enrichment *P*-value of <0.05 are reported. We also calculated false discovery rates (FDRs) for each functional gene set, which represent the estimated probability that a given enrichment score represents a false-positive finding. We only report gene sets with an FDR of <0.25. With such an FDR, the results are likely to be valid at least three out of four times, which is considered a suitable cutoff for the generation of interesting hypotheses (Gu *et al*, 2007). The GSEA was executed using the GSEA 2.0 software package (Broad Institute of MIT and Harvard, Cambridge, MA, USA).

### Leading-edge subset analysis

The leading-edge subset is defined as the subset of genes in a functional gene set that appears in the ranked list of 15 909 genes at, or before, the point in which the running enrichment score reaches its maximum deviation from zero. The genes within this subset can be interpreted as the most important in the enrichment of the functional gene set. Leading-edge subsets were defined for all statistically enriched function gene sets (*P*<0.05). Subsequently, overlap between leading-edge subsets from significantly enriched functional gene sets identified in the different databases was determined to discover genes belonging to more than one leading-edge subset, that is, possible key genes.

### Prognostic value of identified genes and pathways

Genes identified as differentially expressed between high and low TIL-containing samples were correlated with disease-specific survival (DSS) in a large cohort of 157 late-stage ovarian cancer patients previously profiled at our institution (Crijns *et al*, 2009). The log expression levels of individual genes were entered into a univariate Cox proportional hazards regression model. Genes with a *P*<0.05 were considered to be associated with DSS. Furthermore, GSEA was performed on the cohort of 157 ovarian cancers to evaluate associations between DSS and identified pathways.

### Immunohistochemistry

Protein expression of selected enriched genes from identified pathways was evaluated by immunohistochemistry using tissue microarray sections. Tissue microarrays were constructed from paraffin-embedded tumour tissue obtained at primary debulking surgery, performed by the gynaecological oncologists from the University Medical Center Groningen between May 1985 and April 2003. The tissue microarrays contain four 0.6 mm core biopsies from each of 361 patients. For this study, tissue samples obtained at primary debulking surgery from 108 advanced-stage serous ovarian cancer patients were analysed, for whom staining of TILs and HLA-A and HLA-B/C has previously been performed and published (Leffers *et al*, 2009a, 2009b). In addition, staining was performed for HLA-DP/DQ/DR (clone CR3/43, DAKO, Heverlee, Belgium). In brief, after dewaxing and rehydration, 4 *μ*m sections were microwaved in 10 mM citrate buffer pH 6.0 for antigen retrieval. Subsequently, sections were incubated overnight with the primary antibody (dilution 1 : 100). Sections were subsequently incubated with DAKO Envision+ (DAKO). Antigen–antibody reactions were visualised with 3,3-diaminobenzidine. Tissue was counterstained with haematoxylin.

Sections were scored independently by two observers (MG/NL) who were unaware of clinicopathological characteristics and the TIL status of patients. A semiquantitative quality control system was used taking into account both the intensity of staining and the percentage of positive tumour cells as previously described (Leffers *et al*, 2009b). The sum of both scores was used to identify three categories of expression: no expression (total score 0–2), positive expression in a proportion of cells or weak expression in all cells (total score 3–6), and positive expression in a majority of cells (total score 7 and 8). Only patients for whom at least two evaluable cores were available were included for further analysis.

Associations between CD8^+^ TIL and protein expression of selected enriched genes in identified pathways were evaluated using the Jonckheere–Terpstra test (a form of the Kruskal–Wallis test that also tests for linearity). The Mann–Whitney *U-*test was used to evaluate associations between CD8^+^ TIL and well-known prognostic factors (i.e., age, FIGO stage, histological grade, and residual tumour after primary debulking surgery). Disease-specific survival was defined as the date of surgery until the date of death because of ovarian cancer (including fatal complications of treatment) or the date of last follow-up. Differences in DSS based on protein expression levels were plotted using Kaplan–Meier survival curves and evaluated using log-rank tests. SPSS software package for Windows, version 16.0 (SPSS Inc., Chicago, IL, USA) was used. *P*-values of <0.05 were considered statistically significant.

## Results

### Patient characteristics

**Figure 1:**
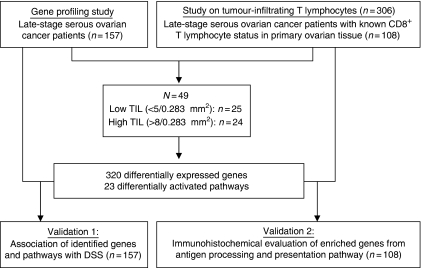
Flowchart illustrating patient selection and validation. Index and validation patients were selected from previous studies at our institute investigating the prognostic effect of gene expression (Crijns *et al*, 2009) and tumour-infiltrating lymphocytes on ovarian cancer patients (Leffers *et al*, 2009a).

Microarray data of 49 advanced-stage serous ovarian cancer patients with either low (*n*=25) or high numbers of CD8^+^ TILs (*n*=24) were evaluated (Figure 1). Clinical and pathological characteristics as well as tumour percentage of the samples used for microarray did not differ between patients with low or high numbers of CD8^+^ TILs (Table 1). Median DSS was higher for patients with high CD8^+^ TILs (log-rank test *P*=0.025).

### Differential expression of genes and biological pathway analysis

A comparison of the expression levels of all 15 909 genes showed differential expression of 320 genes between tumours containing low and high numbers of CD8^+^ TILs (*P*<0.05). The differences between low and high CD8^+^ TIL samples were small (Supplementary Table 1). In view of these small differences, we subsequently evaluated whether coordinated differences in genes belonging to a single biological pathway existed rather than differences in the expression levels of single genes. In all, 14 pathways in BIOCARTA and 8 pathways in KEGG (*P*<0.05, FDR <0.25) were enriched in tumour samples with high numbers of CD8^+^ TILs, whereas only one pathway was enriched in tumour samples with low CD8^+^ TIL numbers (Table 2). Interestingly, and conveniently serving as an internal control, one of the pathways enriched in high CD8^+^ TIL tumours was the CTL-mediated immune response pathway. We subsequently performed leading-edge subset analysis to identify key regulatory genes common to the enriched pathways (Table 3), which among others identified several genes encoding for HLA molecules.

### Effect on DSS

**Figure 2:**
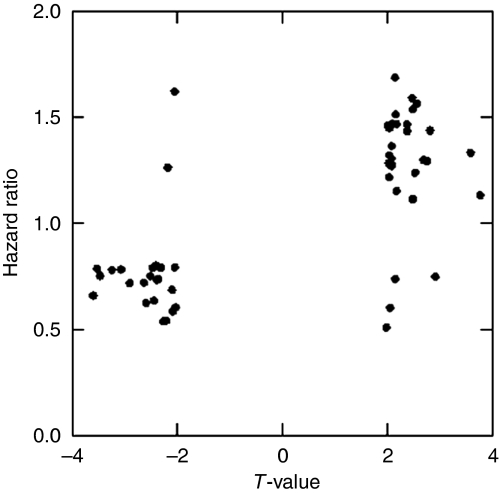
Scatterplot illustrating clustering of differentially expressed genes with survival. Genes with *T*-value <0.0 are differentially expressed in high CD8^+^ TIL tumours, and genes with *T*-value >0.0 are differentially expressed in low CD8^+^ TIL tumours. Hazard ratio <1.0 shows increased survival, whereas hazard ratio >1.0 shows worse survival.

**Figure 3:**
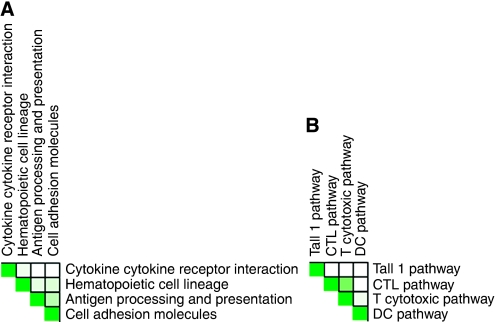
Heatmaps showing interdependency of pathways associated with disease-specific survival and CD8^+^ T lymphocyte infiltration. (**A**) Pathways from KEGG database and (**B**) pathways from BIOCARTA database.

Univariate survival analysis was performed to assess the prognostic value of the 320 genes identified as differentially expressed between high and low CD8^+^ TIL tumours, using 157 late-stage serous ovarian cancer patients previously profiled at our institute (Crijns *et al*, 2009). A significant association with DSS was observed for 54 genes (Table 4). Of the 23 genes associated with high CD8^+^ TIL tumours, 21 were associated with improved survival. Conversely, 27 of 31 genes associated with low CD8^+^ TIL tumours were associated with decreased survival (Figure 2). We next performed GSEA to identify which pathways were associated with DSS. Eight pathways associated with the presence of CD8^+^ TIL, such as the antigen processing and presentation pathway, were also positively associated with DSS (Table 2). The interdependency of these pathways has been visualised in Figure 3A and B.

### Immunohistochemical validation

On the basis of GSEA and leading-edge subset analysis, which showed that the presence of TILs was associated with the expression of MHC class I and II genes as part of the antigen processing and presentation pathway, we evaluated immunohistochemical staining of HLA-A, HLA-B/C, and HLA-DP/DQ/DR in 108 advanced-stage serous ovarian cancer patients for whom information on CD8^+^ TILs was available, part of which was previously published for a larger patient cohort (Leffers *et al*, 2009a, 2009b). Partial or total loss of HLA-A and HLA-B/C was observed in 70.4 and 62.0% of patients, respectively, whereas HLA-DP/DQ/DR upregulation was observed in 68.5% of patients. Increasing levels of MHC class I and II protein expression strongly correlated with increased numbers of CD8^+^ TILs (Table 5). However, no association was observed between the expression of HLA-A, HLA-B/C, and HLA-DP/DQ/DR with DSS (*P*=0.114, *P*=0.599, and *P*=0.692, respectively).

## Discussion

Although it has been well established that TILs are predictors of DSS in ovarian cancer, it is largely unknown what factors contribute to the presence of these lymphocytes. By comparing gene expression profiles of 25 tumours containing low and 24 tumours containing high numbers of CD8^+^ TILs, we identified 320 genes differentially expressed by primary tumours of late-stage serous ovarian cancer patients. In addition, for 54 of these genes, an association with survival was observed in a large validation cohort containing 157 advanced-stage serous ovarian cancer patients. Genes connected to high CD8^+^ TIL tumours were associated with improved survival. With GSEA, next to pathways merely reflecting the presence of lymphocytes, several pathways were identified to be associated with the (lack of) CD8^+^ TILs, some of which were also associated with survival in our validation set. Lastly, the association of a number of genes from the enriched antigen processing and presentation pathway with the presence of CD8^+^ TIL was confirmed using immunohistochemistry.

Our study illustrates that gene expression profiling is a valuable approach to elucidate what tumour cell characteristics contribute to or impede recruitment of lymphocytes into serous ovarian cancer. However, a problem inherent to the design of our study is the impossibility to discern what genes were expressed by tumour cells and what signal was derived from TILs themselves. This is substantiated by the fact that some of the pathways found to be enriched in high CD8^+^ TIL tumours are lymphocyte-specific pathways, for example, T cytotoxic pathway. To our advantage, this observation can also be regarded to validate the immunohistochemical evaluation of CD8^+^ T-cell count used for patient selection. To avoid the signal from lymphocytes, one should profile a limited number of tumour cells, which could be accomplished by microdissection of tumour cells (Glanzer and Eberwine, 2004). However, as with decreasing cell numbers, RNA yield also reduces such assays heavily depend on mRNA amplification. Especially genes with low numbers of transcripts may be under-represented after amplification and thus not identified in subsequent profiling studies (Nygaard *et al*, 2005). For this study, we therefore decided that, as the percentage of tumour cells did not differ between samples with low and high numbers of CD8^+^ TILs, it was safe to assume that differences in non-lymphocyte-restricted genes and pathways reflected differences in gene expression by tumour cells.

Ultimately, our study was intended not only to establish what tumour factors contribute to lymphocyte recruitment, but also to discover putative factors that might enhance clinical results of immunotherapy for ovarian malignancies by improving lymphocyte recruitment when targeted. In this respect, one of the interesting genes identified as differentially expressed between high CD8^+^ TIL and low CD8^+^ TIL tumours and associated with DSS of late-stage serous ovarian cancer patients is interferon regulatory factor 1 (*IRF-1*). Recently, *IRF-1* was reported to be a positive prognostic factor in ovarian and colorectal cancer (Galon *et al*, 2006; Zeimet *et al*, 2009) and was found to be associated with infiltration of CD8^+^ T lymphocytes in ovarian cancer (Callahan *et al*, 2008). Binding of IFN-*γ* to the IFN-*γ* receptor leads to upregulation of *IRF-1*, which in turn results in: (1) induction of IFN-*γ-*inducible genes, such as the *TAP1*, *LMP2*, and *β*_2_-microglobulin genes of the MHC class I-dependent pathway, as well as (2) activation of *CIITA*, a critical transcription factor for MHC class II gene expression. Thus, IRF-1 facilitates recognition of tumour cells by immune cells, ultimately resulting in an IFN-*γ-*dependent positive feedback loop. Correspondingly, in high CD8^+^ TIL tumours we observed differential expression of several MHC class I and II genes, all part of the selectively activated antigen processing and presentation pathway. Immunohistochemical evaluation confirmed the positive association of intra-tumoural cytotoxic T cells with surface expression of MHC class I and II molecules HLA-A, HLA-B/C, and HLA-DP/DQ/DR. Although several MHC class II alleles were differentially expressed in high CD8^+^ TIL tumours, no association was found between HLA-DP/DQ/DR membrane expression and survival. Moreover, only *HLA-DQB2*, a virtually non-polymorphic gene, was associated with DSS in our large validation cohort (Gaur *et al*, 1992). As it encodes six thus far unknown putative proteins, the association of *HLA-DQB2* with CD8^+^ TILs and survival is intriguing and deserves further investigation.

Although we previously reported decreased survival for ovarian cancer patients in association with HLA-B/C downregulation, an association with survival was observed neither at mRNA nor protein level in this study (Leffers *et al*, 2008). The difference in prognostic effect observed in this study could be explained by differences in study population and/or size. In this study a homogeneous population of 108 late-stage serous ovarian cancer patients was used for immunohistochemical validation. Renewed analysis of the previously published data using only late-stage serous ovarian cancer patients (*n*=151) did not yield a correlation of HLA-B/C expression with DSS either (data not shown).

To our knowledge, only one study attempting a better understanding of mechanisms underlying the infiltration by CTLs of serous ovarian cancer by gene profiling of tumour samples has previously been published (Callahan *et al*, 2008), although the genetic signature of ovarian cancer containing high numbers of regulatory T cells was recently published (Barnett *et al*, 2010). It seems that here too genes of the antigen presentation pathway have a major role. Three genes seem to be important for both recruitment of regulatory FoxP3^+^ and cytotoxic CD8^+^ lymphocytes, that is, *CCL5, APOL6*, and *IRF-1*. In the study of Callahan *et al* (2008) that profiled 38 high-grade advanced-stage ovarian carcinomas, 81 genes were associated with CD8^+^ T-cell infiltrate. Only two of these genes, *IRF-1* and *CXCR6*, were also found to be differentially expressed in our cohort of 49 serous advanced-stage ovarian cancer patients (*P*=0.018578 resp. *P*=0.035424). Several possible explanations for the lack of concordance in identified genes exist. Technique-specific issues, including choice of microarray platform and randomisation of samples on arrays, may impede overlap in results (Draghici *et al*, 2006). Furthermore, differences in patient population exist between the two studies, for example, only high-grade tumours *vs* low and high-grade tumours in our study. An additional difficulty, inherent to microarray studies, is the use of small patient cohorts to evaluate large numbers of potential predictors of lymphocyte recruitment. This raises the likelihood of finding distinctive patterns based on chance rather than on biology, a phenomenon called overfitting (Fehrmann *et al*, 2007). Overfitting reduces the chances of finding overlap on the level of individual genes between studies, which can be countered by paying more attention to overlap in functional gene sets rather than individual genes (Crijns *et al*, 2009).

Despite these challenges, the expression of IRF-1, described above, and of CXCR6 was positively associated with the presence of CD8^+^ TILs in both studies. Recently, it was established that both the chemokine receptor CXCR6 and its ligand CXCL16 are not only expressed by immune cells, but also by carcinomas (Meijer *et al*, 2008). Moreover, radiation was shown to recruit lymphocytes to carcinomas through the release of CXCL16 by tumour cells (Matsumura *et al*, 2008). Whether the expression of CXCR6 is similarly induced by radiation and/or chemotherapy and also influences lymphocyte attraction remains to be investigated.

Not only was there a discrepancy between identified genes between our study and that of Callahan *et al* (2008), we also found a substantial difference in genes and pathways associated with survival between the present and our previous study (Crijns *et al*, 2009). An important reason for this divergence is a difference in approach. Whereas the association with survival was the primary focus of our previous study, the current study was designed to discover genes and pathways that might be linked to lymphocyte recruitment. Only identified genes and pathways were subsequently evaluated for an association with survival. A second, although related, reason is the fact that for the present study we were less stringent in the selection of genes (*P*<0.05), as we were primarily interested in associations with lymphocyte recruitment rather than survival, although for the 86-gene profile only genes meeting a *P*<0.001 were selected (Crijns *et al*, 2009). Thus, only two genes associated with survival in the current study were also part of the 86-gene profile, that is, *BRSK1* and *C1orf151*. Neither gene has so far been further investigated to explain its prognostic effect and/or role in lymphocyte recruitment.

In summary, this study shows that gene expression profiling and pathway analysis are valuable strategies to obtain more insight into what tumour characteristics contribute to lymphocyte recruitment to advanced-stage serous ovarian carcinomas. Identified genes and pathways need to be further validated and evaluated for their value as a therapeutic target.

## Figures and Tables

**Table 1:** Clinicopathological characteristics and survival data of patients with known CD8^+^ TIL status

	**Patients in microarray analyses**			**IHC validation set**
	**Low TIL (*n*=25)**	**High TIL (*n*=24)**	***P*-value**	**(*n*=108)**
*Age (years)*				
Mean (s.d.)	62.4 (14.5)	60.8 (12.9)	NS^*^	60.2 (13.2)
				
*DSS (months)*				
Median (95% CI)	10.8 (8.7–13.0)	19.4 (1.0–37.7)	0.025	19.9^a^ (8.4–31.3)
				
*FIGO stage*				
Stage III	19 (76.0%)	20 (83.3%)	NS	87 (80.6%)
Stage IV	6 (24.0%)	2 (16.7%)		21 (19.4%)
				
*Tumour grade*				
Grade I	1 (4.0%)	2 (8.3%)	NS	6 (5.6%)
Grade II	10 (40.0%)	5 (20.8%)		28 (25.9%)
Grade III/undifferentiated	13 (52.0%)	16 (66.7%)		67 (62.0%)
Missing	1 (4.0%)	1 (4.2%)		7 (6.5%)
				
*Residual disease*				
<2 cm	5 (20.0%)	6 (25.0%)	NS	33 (30.6%)
⩾2 cm	20 (80.0%)	16 (66.7%)		66 (61.1%)
Missing	—	2 (8.3%)		9 (8.3%)
				
*Type of chemotherapy*				
No chemotherapy	3 (12.0%)	1 (4.2%)	NS	6 (5.6%)
Platinum containing	11 (44.0%)	12 (50.0%)		41 (38.0%)
Platinum and taxane containing	8 (32.0%)	8 (33.3%)		48 (44.4%)
Other	2 (8.0%)	3 (12.5%)		10 (9.3%)
Missing	1 (4.0%)	—		3 (2.8%)
				
*CD8* ^+^				
Median (IQR)	1.0 (0.5–2.5)	24.0 (16.0–62.5)	<0.001	8.0 (2.0–22.8)
				
% *Tumour in microarray sample*				
Median (IQR)	63 (50.0–80.0)	70 (52.5–78.0)	NS	

Abbreviations: DSS=disease-specific survival; FIGO=International Federation of Gynaecology and Obstetrics; CI=confidence interval; IHC=immunohistochemistry; NS=not significant; IQR=interquartile range; TIL=tumour-infiltrating lymphocyte.

a Only two patients died from other causes than ovarian cancer or treatment-related fatalities.

^*^P⩾0.05.

**Table 2:** Enriched pathways in tumour samples with high CD8^+^ TIL identified by gene set enrichment analysis using pathway definitions from BIOCARTA and KEGG

**Pathway**	**Database**	***P*-value**	**FDR**	**Enriched in**
Ribosome pathway	KEGG	0.0	0.0	Low CD8+ TIL
Antigen processing and presentation^a^	KEGG	0.0	0.0	High CD8+ TIL
Type I diabetes mellitus	KEGG	0.0	0.006	High CD8+ TIL
Toll like receptor signalling pathway	KEGG	0.0	0.055	High CD8+ TIL
Haematopoietic cell lineage^a^	KEGG	0.0	0.060	High CD8+ TIL
Cytokine cytokine receptor interaction^a^	KEGG	0.0	0.104	High CD8+ TIL
Cell adhesion molecules^a^	KEGG	0.0	0.137	High CD8+ TIL
Citrate cycle	KEGG	0.013	0.156	High CD8+ TIL
Reductive carboxylate cycle	KEGG	0.038	0.142	High CD8+ TIL
CTL pathway^a^	BIOCARTA	0.0	0.051	High CD8+ TIL
COMP pathway	BIOCARTA	0.0	0.008	High CD8+ TIL
MITOCHONDRIA pathway	BIOCARTA	0.0	0.120	High CD8+ TIL
D4GDI pathway	BIOCARTA	0.005	0.105	High CD8+ TIL
AMI pathway	BIOCARTA	0.005	0.225	High CD8+ TIL
DC pathway^a^	BIOCARTA	0.007	0.215	High CD8+ TIL
TALL1 pathway^a^	BIOCARTA	0.010	0.201	High CD8+ TIL
ATRBRCA pathway	BIOCARTA	0.017	0.205	High CD8+ TIL
CSK pathway	BIOCARTA	0.024	0.195	High CD8+ TIL
CASPASE pathway	BIOCARTA	0.029	0.183	High CD8+ TIL
STEM pathway	BIOCARTA	0.037	0.230	High CD8+ TIL
T CYTOTOXIC PATHWAY^a^	BIOCARTA	0.038	0.198	High CD8+ TIL
ATM pathway	BIOCARTA	0.038	0.198	High CD8+ TIL
LAIR pathway	BIOCARTA	0.046	0.231	High CD8+ TIL

Abbreviations: TIL=tumour-infiltrating lymphocyte; FDR=false discovery rate; KEGG=Kyoto Encyclopedia of Genes and Genomes.

a Also associated with improved survival in 157 previously profiled advanced-stage ovarian cancer patients (Crijns *et al*, 2009).

**Table 3:** Results of leading-edge analysis identifying genes common to enriched pathways

**KEGG**		**BIOCARTA**	
**Gene symbol**	***N* gene sets**	**Gene symbol**	***N* gene sets**
*HLA-A*	4	*CD3D*	5
*HLA-B*	4	*ITGBB2*	3
*HLA-DRB1*	4	*CD3E*	4
*HLA-DRB2*	4	*HLA-DRB1*	3
*IL1B*	4	*CFS2*	3
*TNFA1*	4	*IL5*	3
*HLA-DQB1*	3	*IL8*	3
*HLA-DQB2*	3	*CD8A*	3
*HLA-F*	3	*CYCS*	3
*CD8A*	3	*GZMB*	3
*NFKB1A*	3	*PRF1*	3
*RELA*	3	*RELA*	3
*IKBKB*	3		
*PIK3R3*	3		
*CSF2*	3		
*IL8*	3		
*CCL5*	3		
*CD40*	3		

Abbreviation: KEGG=Kyoto Encyclopedia of Genes and Genomes.

**Table 4:** Genes differentially expressed between high and low CD8^+^ TIL tumours that are associated with DSS in a large cohort of 157 advanced-stage serous ovarian carcinomas

**Gene symbol**	**Description**	***T***	**Parametric *P*-value lymphoc.**	**Parametric *P*-value survival**	**HR**	**Fold change high/low CD8^+^ TIL**
*ENDOG*	*Endonuclease G*	−3.58818	0.000469	0.007736	0.657	1.057368
*APOL6*	*Apolipoprotein L 6*	−3.51777	0.000588	0.016474	0.784	1.061243
*LOC144817*	*Hypothetical protein loc144817*	−3.4572	0.000725	0.007082	0.751	1.054609
*TNFRSF11B*	*Tumour necrosis factor receptor superfamily, member 11b (osteoprotegerin)*	−3.2365	0.001533	0.029561	0.778	1.076219
*CCL5*	*Chemokine (c-c motif) ligand 5*	−3.05761	0.002678	0.008189	0.78	1.06113
*SMARCD3*	*SWI/SNF-related, matrix-associated, actin-dependent regulator of chromatin, subfamily d, member 3*	−2.89433	0.004415	0.012281	0.715	1.044497
*HLA-DQB2*	*Major histocompatibility complex, class II, DQ β 1*	−2.62341	0.009683	0.025419	0.718	1.036787
*P2RY2*	*Purinergic receptor P2Y, G-protein coupled, 2*	−2.58765	0.010682	0.04794	0.622	1.062139
*HTATIP2*	*HIV-1 tat interactive protein 2, 30kda*	−2.50033	0.013646	0.023893	0.749	1.04496
*ITGB4*	*Integrin, β 4*	−2.4564	0.015268	0.02019	0.788	1.042571
*BRSK1*	*BR serine/threonine kinase 1*	−2.4344	0.016188	0.001032	0.634	1.043443
*CD74*	*CD74 antigen (invariant polypeptide of major histocompatibility complex, class II antigen-associated)*	−2.39153	0.018114	0.048429	0.798	1.0387
*IRF1*	*Interferon regulatory factor 1*	−2.38234	0.018578	0.018766	0.731	1.039269
*CARD9*	*Caspase recruitment domain family, member 9*	−2.34654	0.020354	0.012632	0.733	1.053982
*GBP5*	*Guanylate binding protein 5*	−2.29852	0.023022	0.026438	0.789	1.053155
*OR4K1*	*Olfactory receptor, family 4, subfamily k, member 1*	−2.25082	0.025956	0.027515	0.535	1.043061
*IDH3A*	*Isocitrate dehydrogenase 3 (nad*+*) α*	−2.19777	0.029762	0.004543	0.539	1.042238
*RARRES3*	*Retinoic acid receptor responder (tazarotene induced) 3*	−2.17032	0.031671	0.027127	1.26	1.039329
*GUCY2F*	*Guanylate cyclase 2f, retinal*	−2.09159	0.038394	0.030464	0.683	1.064628
*C1orf151*	*Chromosome 1 open reading frame 151*	−2.08091	0.039363	0.000145	0.582	1.037702
*PUM2*	*Pumilio homologue 2 (drosophila)*	−2.04334	0.042905	0.037206	1.617	1.026572
*SUSD3*	*Sushi domain containing 3*	−2.04205	0.043025	0.0087	0.791	1.041484
*GRM3*	*Glutamate receptor, metabotropic 3*	−2.02279	0.045086	0.007333	0.602	1.037417
*RP9*	*Retinitis pigmentosa 9 (autosomal dominant)*	1.979686	0.049732	0.02947	0.506	0.970948
*TPM2*	*Tropomyosin 2 (β)*	2.01335	0.046075	0.002014	1.459	0.968061
*CREB3L4*	*cAMP responsive element binding protein 3-like 4*	2.024371	0.044851	0.024523	1.448	0.969375
*ADFP*	*Adipose differentiation-related protein*	2.028392	0.044437	0.004516	1.279	0.954856
*ZIC1*	*Zic family member 1 (odd-paired homologue, drosophila)*	2.034892	0.043757	0.024456	1.214	0.941812
*TCF4*	*Transcription factor 4*	2.038027	0.043448	0.041311	1.317	0.965471
*RKHD1*	*Ring finger and KH domain containing 1*	2.054802	0.041812	0.026765	0.6	0.971522
*AKAP12*	*A kinase (PRKA) anchor protein (gravin) 12*	2.05607	0.041754	0.020387	1.448	0.965955
*CENPF*	*Centromere protein f, 350/400 ka (mitosin)*	2.079257	0.039457	0.017257	1.28	0.955777
*TF*	*Transferrin*	2.085059	0.039044	0.019715	1.302	0.95165
*MARCKS*	*Myristoylated alanine-rich protein kinase c substrate*	2.085433	0.038849	0.01913	1.361	0.967259
*FBN3*	*Fibrillin 3*	2.08694	0.038733	0.008793	1.266	0.942574
*TSPAN13*	*Tetraspanin 13*	2.102597	0.037447	0.00351	1.465	0.962836
*C1orf85*	*Chromosome 1 open reading frame 85*	2.149681	0.033301	0.009093	1.684	0.969927
*NT5C2*	*5′-nucleotidase, cytosolic II*	2.151955	0.033136	0.042652	0.735	0.959484
*DAD1*	*Defender against cell death 1*	2.163076	0.032559	0.039308	1.509	0.971288
*COL11A1*	*Collagen, type xi, α 1*	2.180716	0.03094	0.008437	1.149	0.926402
*GLT25D1*	*Glycosyltransferase 25 domain containing 1*	2.183846	0.030707	0.011982	1.464	0.966298
*GMFB*	*Glia maturation factor, β*	2.381146	0.018725	0.011898	1.432	0.962138
*SNRPE*	*Small nuclear ribonucleoprotein polypeptide e*	2.38174	0.018656	0.012262	1.462	0.983812
*ZNF281*	*Zinc-finger protein 281*	2.473985	0.014571	0.023546	1.587	0.957854
*FABP4*	*Fatty acid binding protein 4, adipocyte*	2.485825	0.014192	0.025061	1.11	0.908835
*SAE2*	*SUMO1 activating enzyme subunit 2*	2.489129	0.014051	0.018709	1.535	0.965225
*BASP1*	*Brain abundant, membrane attached signal protein 1*	2.533806	0.012459	0.032678	1.235	0.94384
*ARMCX3*	*Armadillo repeat containing, x-linked 3*	2.563703	0.011458	0.006661	1.562	0.94847
*CPVL*	*Carboxypeptidase, vitellogenic-like*	2.690174	0.008038	0.048239	1.295	0.957396
*FMOD*	*Fibromodulin*	2.76574	0.006462	0.012624	1.289	0.945678
*C3orf59*	*Chromosome 3 open reading frame 59*	2.822682	0.005501	0.022204	1.433	0.950026
*PXDN*	*Peroxidasin homologue (drosophila)*	2.920898	0.004082	0.049685	0.746	0.94662
*MEST*	*Mesoderm specific transcript homologue (mouse)*	3.590373	0.000463	0.002053	1.328	0.940516
*HMGA2*	*High mobility group at-hook 2*	3.770553	0.00024	0.031783	1.128	0.886346

Abbreviations: DSS=disease-specific survival; TIL=tumour-infiltrating lymphocyte; HR=hazard ratio.

**Table 5:** Association of HLA protein expression evaluated by immunohistochemistry with CD8^+^ TILs in serous advanced-stage ovarian cancer

		**CD8^+^ T lymphocytes**			
	***N* (%)**	**M**	**IQR**	***Z***	***P*-value^a^**
*HLA-A*					
Total loss	27 (25.0)	1.0	1.0–12.0	3.96	<0.001
Partial loss	49 (45.4)	9.0	4.0–22.0		
Normal expression	32 (29.6)	14.0	4.8–55.3		
					
*HLA-B/C*					
Total loss	23 (21.3)	2.0	1.0–15.0	4.19	<0.001
Partial loss	44 (40.7)	6.0	2.0–19.8		
Normal expression	41 (38.0)	16.0	5.5–30.5		
					
*HLA-DP/DQ/DR*					
No expression	34 (31.5)	2.0	1.0–11.0	3.36	0.001
Upregulation	52 (48.1)	12.0	5.0–24.0		
Strong upregulation	22 (20.4)	14.5	4.5–43.0		

Abbreviations: M=median; IQR=interquartile range; TIL=tumour-infiltrating lymphocyte.

a Calculated using Jonckheere–Terpstra test.

## References

[bib1] Barnett JC, Bean SM, Whitaker RS, Kondoh E, Baba T, Fujii S, Marks JR, Dressman HK, Murphy SK, Berchuck A (2010) Ovarian cancer tumor infiltrating T-regulatory (T(reg)) cells are associated with a metastatic phenotype. Gynecol Oncol 116: 556–562 20006900 10.1016/j.ygyno.2009.11.020

[bib2] Buckanovich RJ, Facciabene A, Kim S, Benencia F, Sasaroli D, Balint K, Katsaros D, O’Brien-Jenkins A, Gimotty PA, Coukos G (2008) Endothelin B receptor mediates the endothelial barrier to T cell homing to tumors and disables immune therapy. Nat Med 14: 28–36 18157142 10.1038/nm1699

[bib3] Callahan MJ, Nagymanyoki Z, Bonome T, Johnson ME, Litkouhi B, Sullivan EH, Hirsch MS, Matulonis UA, Liu J, Birrer MJ, Berkowitz RS, Mok SC (2008) Increased HLA-DMB expression in the tumor epithelium is associated with increased CTL infiltration and improved prognosis in advanced-stage serous ovarian cancer. Clin Cancer Res 14: 7667–7673 19047092 10.1158/1078-0432.CCR-08-0479 PMC3000165

[bib4] Cancer Committee of the International Federation of Gynaecology and Obstetrics (1986) Staging announcement: FIGO Cancer Committee. Gynecol Oncol 25: 383–385

[bib5] Churchill GA (2002) Fundamentals of experimental design for cDNA microarrays. Nat Genet 32(Suppl): 490–495 12454643 10.1038/ng1031

[bib6] Crijns AP, Duiker EW, de Jong S, Willemse PH, van der Zee AG, de Vries EG (2006) Molecular prognostic markers in ovarian cancer: toward patient-tailored therapy. Int J Gynecol Cancer 16(Suppl 1): 152–165 10.1111/j.1525-1438.2006.00503.x 16515584

[bib7] Crijns AP, Fehrmann RS, de Jong S, Gerbens F, Meersma GJ, Klip HG, Hollema H, Hofstra RM, te Meerman GJ, de Vries EG, van der Zee AG (2009) Survival-related profile, pathways, and transcription factors in ovarian cancer. PLoS Med 6: e24 19192944 10.1371/journal.pmed.1000024 PMC2634794

[bib8] Curiel TJ, Coukos G, Zou L, Alvarez X, Cheng P, Mottram P, Evdemon-Hogan M, Conejo-Garcia JR, Zhang L, Burow M, Zhu Y, Wei S, Kryczek I, Daniel B, Gordon A, Myers L, Lackner A, Disis ML, Knutson KL, Chen L, Zou W (2004) Specific recruitment of regulatory T cells in ovarian carcinoma fosters immune privilege and predicts reduced survival. Nat Med 10: 942–949 15322536 10.1038/nm1093

[bib9] Draghici S, Khatri P, Eklund AC, Szallasi Z (2006) Reliability and reproducibility issues in DNA microarray measurements. Trends Genet 22: 101–109 16380191 10.1016/j.tig.2005.12.005 PMC2386979

[bib10] Fehrmann RS, Li XY, van der Zee AG, de Jong S, Te Meerman GJ, de Vries EG, Crijns AP (2007) Profiling studies in ovarian cancer: a review. Oncologist 12: 960–966 17766655 10.1634/theoncologist.12-8-960

[bib11] Galon J, Costes A, Sanchez-Cabo F, Kirilovsky A, Mlecnik B, Lagorce-Pages C, Tosolini M, Camus M, Berger A, Wind P, Zinzindohoue F, Bruneval P, Cugnenc PH, Trajanoski Z, Fridman WH, Pages F (2006) Type, density, and location of immune cells within human colorectal tumors predict clinical outcome. Science 313: 1960–1964 17008531 10.1126/science.1129139

[bib12] Gaur LK, Heise ER, Thurtle PS, Nepom GT (1992) Conservation of the HLA-DQB2 locus in nonhuman primates. J Immunol 149: 2530 1356127

[bib13] Glanzer JG, Eberwine JH (2004) Expression profiling of small cellular samples in cancer: less is more. Br J Cancer 90: 1111–1114 15026786 10.1038/sj.bjc.6601668 PMC2409658

[bib14] Gu Q, Tan M, Sun Y (2007) SAG/ROC2/Rbx2 is a novel activator protein-1 target that promotes c-Jun degradation and inhibits 12-O-tetradecanoylphorbol-13-acetate-induced neoplastic transformation. Cancer Res 67: 3616–3625 17440073 10.1158/0008-5472.CAN-06-4020

[bib15] Hsu JC, Chang J, Wang T, Steingrimsson E, Magnusson MK, Bergsteinsdottir K (2007) Statistically designing microarrays and microarray experiments to enhance sensitivity and specificity. Brief Bioinform 8: 22–31 16899493 10.1093/bib/bbl023

[bib16] Hung CF, Wu TC, Monie A, Roden R (2008) Antigen-specific immunotherapy of cervical and ovarian cancer. Immunol Rev 222: 43–69 18363994 10.1111/j.1600-065X.2008.00622.x PMC2692865

[bib17] Kerr MK (2003) Design considerations for efficient and effective microarray studies. Biometrics 59: 822–828 14969460 10.1111/j.0006-341x.2003.00096.x

[bib18] Leffers N, Gooden MJ, de Jong RA, Hoogeboom BN, ten Hoor KA, Hollema H, Boezen HM, van der Zee AG, Daemen T, Nijman HW (2009a) Prognostic significance of tumor-infiltrating T-lymphocytes in primary and metastatic lesions of advanced stage ovarian cancer. Cancer Immunol Immunother 58: 449–459 18791714 10.1007/s00262-008-0583-5 PMC11030692

[bib19] Leffers N, Gooden MJ, Mokhova AA, Kast WM, Boezen HM, ten Hoor KA, Hollema H, Daemen T, van der Zee AG, Nijman HW (2009b) Down-regulation of proteasomal subunit MB1 is an independent predictor of improved survival in ovarian cancer. Gynecol Oncol 113: 256–263 19243813 10.1016/j.ygyno.2008.12.030

[bib20] Leffers N, Lambeck AJA, de Graeff P, Bijlsma AY, Daemen T, van der Zee AGJ, Nijman HW (2008) Survival of ovarian cancer patients overexpressing the tumour antigen p53 is diminished in case of MHC class I down-regulation. Gynecol Oncol 110: 365–373 18571704 10.1016/j.ygyno.2008.04.043

[bib21] Matsumura S, Wang B, Kawashima N, Braunstein S, Badura M, Cameron TO, Babb JS, Schneider RJ, Formenti SC, Dustin ML, Demaria S (2008) Radiation-induced CXCL16 release by breast cancer cells attracts effector T cells. J Immunol 181: 3099–3107 18713980 10.4049/jimmunol.181.5.3099 PMC2587101

[bib22] Meijer J, Ogink J, Kreike B, Nuyten D, de Visser KE, Roos E (2008) The chemokine receptor CXCR6 and its ligand CXCL16 are expressed in carcinomas and inhibit proliferation. Cancer Res 68: 4701–4708 18559516 10.1158/0008-5472.CAN-08-0482

[bib23] Nygaard V, Holden M, Loland A, Langaas M, Myklebost O, Hovig E (2005) Limitations of mRNA amplification from small-size cell samples. BMC Genomics 6: 147 16253144 10.1186/1471-2164-6-147 PMC1310617

[bib24] Parkin DM, Bray F, Ferlay J, Pisani P (2005) Global cancer statistics, 2002. CA Cancer J Clin 55: 74–108 15761078 10.3322/canjclin.55.2.74

[bib25] Sato E, Olson SH, Ahn J, Bundy B, Nishikawa H, Qian F, Jungbluth AA, Frosina D, Gnjatic S, Ambrosone C, Kepner J, Odunsi T, Ritter G, Lele S, Chen YT, Ohtani H, Old LJ, Odunsi K (2005) Intraepithelial CD8+ tumor-infiltrating lymphocytes and a high CD8+/regulatory T cell ratio are associated with favorable prognosis in ovarian cancer. Proc Natl Acad Sci USA 102: 18538–18543 16344461 10.1073/pnas.0509182102 PMC1311741

[bib26] Scully RE (1999) Histological Typing of Ovarian Tumours. Springer: Berlin

[bib27] World Health Organization (1979) Handbook for Reporting Results of Cancer Treatment. World Health Organization: Geneva

[bib28] Zeimet AG, Reimer D, Wolf D, Fiegl H, Concin N, Wiedemair A, Wolf AM, Rumpold H, Muller-Holzner E, Marth C (2009) Intratumoral interferon regulatory factor (IRF)-1 but not IRF-2 is of relevance in predicting patient outcome in ovarian cancer. Int J Cancer 124: 2353–2360 19170204 10.1002/ijc.24214

[bib29] Zhang L, Conejo-Garcia JR, Katsaros D, Gimotty PA, Massobrio M, Regnani G, Makrigiannakis A, Gray H, Schlienger K, Liebman MN, Rubin SC, Coukos G (2003) Intratumoral T cells, recurrence, and survival in epithelial ovarian cancer. N Engl J Med 348: 203–213 12529460 10.1056/NEJMoa020177

