# Supplementary material for: Identification of genes and pathways associated with cytotoxic T lymphocyte infiltration of serous ovarian cancer
Source: Br J Cancer. 2010 Jul 27;103(5):685–92. doi: 10.1038/sj.bjc.6605820 (PMC2938262; doi:10.1038/sj.bjc.6605820)
Supplement: Supplementary Table 1 [file 6605820x1.doc]

| **Supplementary Table 1:** Differentially expressed genes between high and low TIL tumour samples | | | | | | |
| --- | --- | --- | --- | --- | --- | --- |
| Gene symbol | Parametric  p-value | fold change  (high TIL/low TIL) |  | Gene symbol | Parametric  p-value | fold change  (high TIL/low TIL) |
| LOC440871 | 7,43E-06 | 1,117251 |  | IL2RG | 0,006827 | 1,075053 |
| C18orf16 | 4,97E-05 | 1,148209 |  | OR51I1 | 0,007021 | 1,043944 |
| SCG5 | 0,000131 | 0,923036 |  | TMOD1 | 0,007066 | 1,052378 |
| IGJ | 0,000182 | 1,09965 |  | CCL20 | 0,007176 | 1,082901 |
| CMIP | 0,000183 | 1,067873 |  | TROVE2 | 0,007794 | 0,961688 |
| HMGA2 | 0,00024 | 0,886346 |  | FCMD | 0,007847 | 1,05321 |
| PTEN | 0,000276 | 0,942375 |  | CPVL | 0,008038 | 0,957396 |
| OCLN | 0,000363 | 1,079711 |  | NKG7 | 0,008153 | 1,065044 |
| MEST | 0,000463 | 0,940516 |  | HLA-DRB5 | 0,00818 | 1,022035 |
| ENDOG | 0,000469 | 1,057368 |  | EGLN3 | 0,008322 | 1,064923 |
| LOC650405 | 0,000571 | 1,091009 |  | RPP25 | 0,008593 | 1,056016 |
| APOL6 | 0,000588 | 1,061243 |  | GPR149 | 0,008615 | 1,058556 |
| LOC144817 | 0,000725 | 1,054609 |  | HOXC6 | 0,008647 | 1,035913 |
| SERPINA1 | 0,00073 | 1,0625 |  | HRASLS2 | 0,009056 | 1,056497 |
| LCN2 | 0,001001 | 1,074102 |  | FAM114A1 | 0,009358 | 0,957671 |
| MYH10 | 0,00103 | 0,950461 |  | DOK2 | 0,009436 | 1,037339 |
| BEXL1 | 0,001207 | 0,952302 |  | HLA-DQB2 | 0,009683 | 1,036787 |
| MFAP5 | 0,001248 | 0,929517 |  | AFF2 | 0,009731 | 1,039478 |
| CXCL9 | 0,001499 | 1,109033 |  | BMX | 0,00999 | 1,075003 |
| TNFRSF11B | 0,001533 | 1,076219 |  | INTS1 | 0,010041 | 1,064488 |
| FST | 0,00155 | 0,929508 |  | XPNPEP2 | 0,01009 | 1,076185 |
| INDO | 0,00155 | 1,078666 |  | SFT2D1 | 0,010131 | 0,965259 |
| HPR | 0,001764 | 1,089463 |  | PLS3 | 0,010246 | 0,940982 |
| C16orf24 | 0,001844 | 1,038406 |  | P2RY2 | 0,010682 | 1,062139 |
| CDH2 | 0,002 | 0,953517 |  | EPSTI1 | 0,010933 | 1,049835 |
| CD7 | 0,002024 | 1,084766 |  | ARMCX3 | 0,011458 | 0,94847 |
| CXCL10 | 0,002089 | 1,068514 |  | DNAJC12 | 0,01184 | 0,926049 |
| GBP1 | 0,002201 | 1,069424 |  | RAB11A | 0,011922 | 1,038519 |
| BEX1 | 0,002275 | 0,953609 |  | BASP1 | 0,012459 | 0,94384 |
| OR7D2 | 0,002476 | 1,027473 |  | SOSTDC1 | 0,012824 | 0,948313 |
| SAT1 | 0,002621 | 1,050012 |  | NDN | 0,012829 | 0,948066 |
| CCL5 | 0,002678 | 1,06113 |  | PLXDC2 | 0,013206 | 0,96359 |
| CTSH | 0,002974 | 1,0427 |  | HTATIP2 | 0,013646 | 1,04496 |
| SCLY | 0,003134 | 1,064695 |  | NGFRAP1 | 0,014045 | 0,931023 |
| HRG | 0,003725 | 1,047394 |  | SAE2 | 0,014051 | 0,965225 |
| LOC652745 | 0,003735 | 1,039344 |  | FABP4 | 0,014192 | 0,908835 |
| BEX2 | 0,003791 | 0,959877 |  | TCF7L2 | 0,014303 | 0,955611 |
| PXDN | 0,004082 | 0,94662 |  | HDAC2 | 0,014459 | 0,954354 |
| SMARCD3 | 0,004415 | 1,044497 |  | ZNF281 | 0,014571 | 0,957854 |
| ALCAM | 0,004481 | 0,947927 |  | GDF15 | 0,014646 | 0,952374 |
| HP | 0,004653 | 1,091648 |  | SLC27A6 | 0,01489 | 1,058964 |
| HLA-B | 0,005041 | 1,040748 |  | CD2 | 0,014891 | 1,061113 |
| OPLAH | 0,00505 | 1,071437 |  | EXOSC1 | 0,015039 | 0,963865 |
| C3orf59 | 0,005501 | 0,950026 |  | RPL22L1 | 0,015255 | 0,956496 |
| ZDHHC14 | 0,005513 | 0,944562 |  | ITGB4 | 0,015268 | 1,042571 |
| TNFRSF9 | 0,005694 | 1,103201 |  | TIMP3 | 0,015486 | 0,95625 |
| PTPRK | 0,005893 | 0,956806 |  | DUSP1 | 0,015651 | 0,949952 |
| BST2 | 0,005894 | 1,043544 |  | BRSK1 | 0,016188 | 1,043443 |
| AGR2 | 0,005974 | 0,928906 |  | RAB11FIP2 | 0,016538 | 0,953661 |
| PSMB10 | 0,006215 | 1,05963 |  | XPNPEP1 | 0,016544 | 0,959757 |
| FAHD1 | 0,006308 | 0,953147 |  | DKK1 | 0,01655 | 0,929787 |
| FMOD | 0,006462 | 0,945678 |  | TREX1 | 0,016825 | 1,026756 |

| **Supplementary Table 1** - *continued* | | | | | | |
| --- | --- | --- | --- | --- | --- | --- |
| Gene symbol | Parametric  p-value | fold change  (high TIL/low TIL) |  | Gene symbol | Parametric  p-value | fold change  (high TIL/low TIL) |
| TMSB10 | 0,016857 | 0,982603 |  | OR4K1 | 0,025956 | 1,043061 |
| TLX3 | 0,017642 | 1,059934 |  | EBF1 | 0,026315 | 1,058574 |
| FMO2 | 0,017694 | 0,930059 |  | HES6 | 0,026319 | 1,052962 |
| SERPINH1 | 0,017929 | 0,971745 |  | HMGN3 | 0,026475 | 0,971819 |
| SERPINE1 | 0,017941 | 0,932668 |  | SNHG5 | 0,026532 | 0,961322 |
| CD74 | 0,018114 | 1,0387 |  | C5orf26 | 0,026575 | 0,959577 |
| ATP8B4 | 0,018262 | 1,050496 |  | GZMA | 0,026672 | 1,054404 |
| STXBP3 | 0,018296 | 1,040958 |  | GNL3L | 0,026721 | 1,036245 |
| HSD17B14 | 0,018508 | 0,945075 |  | CTGF | 0,026844 | 0,956754 |
| IRF1 | 0,018578 | 1,039269 |  | TMSL4 | 0,027007 | 0,980662 |
| SNRPE | 0,018656 | 0,966889 |  | ALDH6A1 | 0,027111 | 0,96736 |
| GMFB | 0,018725 | 0,962138 |  | GGT1 | 0,027765 | 0,936654 |
| ECGF1 | 0,019142 | 1,047679 |  | SCG3 | 0,027868 | 0,91115 |
| NUCKS1 | 0,019547 | 0,970186 |  | HOXA7 | 0,029009 | 0,961259 |
| WDR36 | 0,02033 | 0,962181 |  | TXNDC10 | 0,029053 | 0,968704 |
| CARD9 | 0,020354 | 1,053982 |  | GSDMDC1 | 0,029442 | 1,041504 |
| ARL13B | 0,020631 | 0,951318 |  | EIF3S10 | 0,029451 | 0,971334 |
| SFRP2 | 0,020799 | 0,926297 |  | PCDH21 | 0,029458 | 1,05277 |
| CCT6B | 0,020948 | 1,057199 |  | IQCA | 0,02948 | 0,963474 |
| C15orf48 | 0,021006 | 1,075849 |  | ZFP36L1 | 0,029489 | 0,970374 |
| NME3 | 0,02122 | 0,965483 |  | HLA-DRB1 | 0,029529 | 1,031104 |
| SERPINA3 | 0,021544 | 1,04996 |  | GGTL4 | 0,029705 | 1,030813 |
| MYRIP | 0,021668 | 1,040777 |  | IDH3A | 0,029762 | 1,042238 |
| MLKL | 0,021702 | 1,046805 |  | SPOCK1 | 0,029804 | 0,962544 |
| GALNT5 | 0,021746 | 1,044584 |  | TMEM38B | 0,029891 | 1,048449 |
| SLC40A1 | 0,021875 | 1,048227 |  | HYI | 0,029981 | 0,965 |
| HEY2 | 0,021983 | 0,958874 |  | C8orf55 | 0,030142 | 1,028367 |
| MX1 | 0,022039 | 1,046448 |  | TMEM47 | 0,030284 | 0,966829 |
| ATP1A2 | 0,022163 | 0,954493 |  | PRSS22 | 0,03031 | 1,036198 |
| EGR1 | 0,022178 | 0,9673 |  | IRX1 | 0,030573 | 0,956758 |
| ZNF91 | 0,022212 | 0,961726 |  | GLT25D1 | 0,030707 | 0,966298 |
| HOXD10 | 0,022272 | 1,043444 |  | SRPX2 | 0,030712 | 0,95038 |
| TM9SF3 | 0,022464 | 0,963999 |  | COL11A1 | 0,03094 | 0,926402 |
| HSD17B6 | 0,022521 | 0,948245 |  | ZDHHC18 | 0,031512 | 1,030895 |
| JUND | 0,022585 | 0,974256 |  | CCDC43 | 0,031636 | 0,969207 |
| CYP4Z2P | 0,022618 | 1,05979 |  | RARRES3 | 0,031671 | 1,039329 |
| ACTN1 | 0,022886 | 0,970493 |  | ANXA11 | 0,031961 | 0,966047 |
| GBP5 | 0,023022 | 1,053155 |  | MGC24103 | 0,032138 | 0,962419 |
| ARL8A | 0,023184 | 1,03448 |  | SGCB | 0,032169 | 0,961194 |
| GTF2H5 | 0,023662 | 0,964764 |  | FAM112B | 0,032185 | 1,057999 |
| RPS12 | 0,023997 | 0,984371 |  | DAD1 | 0,032559 | 0,971288 |
| PARD3 | 0,02411 | 0,965383 |  | TTYH2 | 0,032791 | 0,948983 |
| LOC339804 | 0,024268 | 1,045769 |  | SIRT6 | 0,032926 | 0,936267 |
| C7orf23 | 0,024385 | 1,038642 |  | NT5C2 | 0,033136 | 0,959484 |
| COL9A3 | 0,02446 | 0,956419 |  | MAGEC1 | 0,033226 | 1,040437 |
| LOC388789 | 0,024749 | 0,969646 |  | C1orf85 | 0,033301 | 0,969927 |
| MAGEA2B | 0,024758 | 1,079554 |  | HPS6 | 0,033599 | 0,971893 |
| TMEM144 | 0,025004 | 1,041673 |  | ZFP42 | 0,033657 | 0,944134 |
| TRIM47 | 0,025151 | 1,045587 |  | SERPINB5 | 0,033664 | 0,9477 |
| RUNX3 | 0,025661 | 1,047044 |  | PPP4R1 | 0,033733 | 1,065693 |
| IL8 | 0,025716 | 1,077473 |  | ITLN1 | 0,033816 | 1,035563 |

| **Supplementary Table 1** - *continued* | | | | | | |
| --- | --- | --- | --- | --- | --- | --- |
| Gene symbol | Parametric  p-value | fold change  (high TIL/low TIL) |  | Gene symbol | Parametric  p-value | fold change  (high TIL/low TIL) |
| TEX264 | 0,034013 | 1,032305 |  | POLR2I | 0,04158 | 0,965969 |
| C10orf71 | 0,034134 | 0,971296 |  | IL1R2 | 0,041643 | 1,031374 |
| HMMR | 0,034151 | 1,06189 |  | AKAP12 | 0,041754 | 0,965955 |
| NDRG2 | 0,034473 | 0,972944 |  | RKHD1 | 0,041812 | 0,971522 |
| BARX1 | 0,034602 | 0,970175 |  | C17orf81 | 0,041819 | 0,95786 |
| GAS5 | 0,035271 | 0,968486 |  | RNMTL1 | 0,041841 | 0,958277 |
| CXCR6 | 0,035424 | 1,039526 |  | SYMPK | 0,041907 | 1,026278 |
| AMOTL2 | 0,035524 | 0,978737 |  | LOC116143 | 0,042157 | 1,036751 |
| PLEKHF2 | 0,035701 | 1,04095 |  | PACRG | 0,042164 | 1,041308 |
| CARHSP1 | 0,035752 | 0,97425 |  | HSD17B11 | 0,042209 | 0,964303 |
| STAT1 | 0,035871 | 1,036775 |  | PUM2 | 0,042905 | 1,026572 |
| TNFRSF17 | 0,035997 | 1,070352 |  | SUSD3 | 0,043025 | 1,041484 |
| CCL18 | 0,036122 | 1,067768 |  | FNDC3B | 0,043043 | 0,967155 |
| KBTBD3 | 0,036206 | 1,06347 |  | CNN1 | 0,04328 | 0,95379 |
| DNMT3A | 0,036243 | 0,963485 |  | TCF4 | 0,043448 | 0,965471 |
| ERVWE1 | 0,036425 | 1,052433 |  | RRM2 | 0,043518 | 0,956725 |
| PRKCI | 0,036571 | 0,96471 |  | B4GALT5 | 0,043699 | 0,966929 |
| RASGRP1 | 0,036672 | 1,044106 |  | ZIC1 | 0,043757 | 0,941812 |
| NUDT17 | 0,036846 | 1,04814 |  | PRELP | 0,044048 | 0,957666 |
| CEBPB | 0,037233 | 0,984643 |  | LTA4H | 0,044071 | 0,972399 |
| GPAM | 0,037345 | 0,9631 |  | ZBTB6 | 0,044176 | 1,036838 |
| TSPAN13 | 0,037447 | 0,962836 |  | GPR31 | 0,044306 | 1,042828 |
| MTMR9 | 0,037733 | 0,961098 |  | CDK5R1 | 0,044415 | 0,969072 |
| C11orf73 | 0,037812 | 0,971325 |  | ADFP | 0,044437 | 0,954856 |
| CYB5A | 0,037822 | 0,97111 |  | ITGA8 | 0,044724 | 0,948782 |
| H1FOO | 0,038312 | 0,968405 |  | AGPAT2 | 0,044728 | 1,031039 |
| COL5A2 | 0,038324 | 0,950367 |  | VPS4B | 0,044807 | 0,96831 |
| LY75 | 0,038389 | 1,055312 |  | CREB3L4 | 0,044851 | 0,969375 |
| GUCY2F | 0,038394 | 1,064628 |  | GRM3 | 0,045086 | 1,037417 |
| TOMM7 | 0,038556 | 0,973502 |  | TAF13 | 0,045274 | 1,065998 |
| FBN3 | 0,038733 | 0,942574 |  | C1orf78 | 0,045286 | 0,975181 |
| MARCKS | 0,038849 | 0,967259 |  | SAMD9L | 0,04532 | 1,047637 |
| TF | 0,039044 | 0,95165 |  | CCDC80 | 0,045385 | 0,961262 |
| SYDE1 | 0,039193 | 0,964285 |  | HSD11B1L | 0,045535 | 0,945436 |
| C1orf151 | 0,039363 | 1,037702 |  | SP140 | 0,045633 | 1,060005 |
| CENPF | 0,039457 | 0,955777 |  | UCK2 | 0,045869 | 0,967043 |
| SEMA5A | 0,039527 | 0,957717 |  | TPM2 | 0,046075 | 0,968061 |
| LDHB | 0,039572 | 0,972471 |  | PPP3CB | 0,046152 | 0,968526 |
| COX15 | 0,03971 | 0,963944 |  | NEUROG3 | 0,046248 | 1,026144 |
| HLA-F | 0,039758 | 1,031955 |  | RPLP0P2 | 0,046301 | 0,982024 |
| ZBTB26 | 0,039949 | 1,042478 |  | CDV3 | 0,046337 | 0,96992 |
| FOSL1 | 0,040064 | 1,027777 |  | C11orf71 | 0,046529 | 1,027314 |
| DHRS9 | 0,040106 | 1,047801 |  | NLRP7 | 0,047212 | 0,94897 |
| FAM84A | 0,040423 | 0,969105 |  | FGB | 0,047212 | 0,930181 |
| IRF4 | 0,040648 | 1,054497 |  | BZRPL1 | 0,047361 | 0,968624 |
| LAG3 | 0,040656 | 1,051308 |  | UQCRC1 | 0,047506 | 1,028321 |
| RBBP7 | 0,040772 | 1,02828 |  | SLC2A8 | 0,047528 | 1,037773 |
| CAMK1G | 0,040862 | 1,059712 |  | RPL23A | 0,047784 | 0,982168 |
| M6PRBP1 | 0,041344 | 0,971057 |  | ZNF307 | 0,047875 | 0,976348 |
| RPS13 | 0,041466 | 0,992434 |  | OR5D14 | 0,048091 | 1,029101 |
| ATP6V1D | 0,04149 | 0,970117 |  | ERC1 | 0,048124 | 0,954965 |

| **Supplementary Table 1** - *continued* | | | | | | |
| --- | --- | --- | --- | --- | --- | --- |
| Gene symbol | Parametric  p-value | fold change  (high TIL/low TIL) |  | Gene symbol | Parametric  p-value | fold change  (high TIL/low TIL) |
| SNX9 | 0,048329 | 0,958665 |  | HSP90AA1 | 0,049175 | 0,976061 |
| CHRAC1 | 0,048371 | 1,039782 |  | LRRFIP2 | 0,049324 | 1,027732 |
| AK3 | 0,048466 | 1,037551 |  | ACAA2 | 0,04962 | 0,967112 |
| IL32 | 0,0486 | 1,027019 |  | ZNF277P | 0,049645 | 0,967039 |
| NAT1 | 0,048792 | 1,039456 |  | CENPK | 0,049656 | 1,040088 |
| TTLL12 | 0,048986 | 1,070338 |  | RP9 | 0,049732 | 0,970948 |
